# Two-stage total joint replacement for hip or knee septic arthritis: post-traumatic etiology and difficult-to-treat infections predict poor outcomes

**DOI:** 10.1007/s00402-024-05249-x

**Published:** 2024-03-02

**Authors:** Antonio Russo, Filippo Migliorini, Fortunato Giustra, Francesco Bosco, Alessandro Massè, Giorgio Burastero

**Affiliations:** 1 Centro Traumatologico Ortopedico (CTO), Department of Orthopaedic Surgery, University of Turin, Via Gianfranco Zuretti, 29 10126, Turin, Italy; 2 Department of Orthopaedic, Trauma, and Reconstructive Surgery, RWTH University Medical Centre, 52074 Aachen, Germany; 3 Department of Orthopedics and Trauma Surgery, Academic Hospital of Bolzano (SABES-ASDAA), Bolzano, 39100 Italy; 4 Department of Orthopaedics and Traumatology, Ospedale San Giovanni Bosco di Torino - ASL Città di Torino, Turin, Italy; 5 Department of Precision Medicine in Medical, Surgical and Critical Care (Me.Pre.C.C.), University of Palermo, Palermo, Italy; 6 Department of Orthopaedics and Traumatology, G.F. Ingrassia Hospital Unit, ASP 6, Palermo, Italy; 7 IRCCS Ospedale Galeazzi-Sant’Ambrogio, Via Cristina Belgioioso 173, Milan, 20157 Italy

**Keywords:** Septic arthritis, Hip, Knee, Two-stage, Treatment, Total joint replacement

## Abstract

**Purpose:**

Septic arthritis (SA) is a rare but significant clinical challenge in orthopedics that can impact patients’ quality of life. This study aims to examine the clinical outcomes of patients undergoing two-stage total joint replacement (TJR) in hip and knee SA and analyze potential predictors of treatment failure.

**Methods:**

A retrospective analysis was conducted using data from a prospectively collected institutional arthroplasty registry from January 1st, 2012, to January 1st, 2019. Patients with hip or knee SA who underwent a two-stage TJR and had at least two years of follow-up were included. Demographic characteristics, surgical variables, and outcomes were collected and analyzed from clinical and surgical data. Statistical analysis was performed using IBM SPSS Statistics, with statistical significance at *p* < 0.05.

**Results:**

One hundred and fourteen patients (61 with hip SA, 53 with knee SA) were included in the study. The mean follow-up was 72.8 months. Postoperatively, both clinical and functional outcomes significantly improved, as indicated by the Hip Society Score (HHS) and Knee Society Score (KSS). The overall success rate of the two-stage protocol was 89.5%. Complications that did not require revision occurred in 21% of cases. The most identified pathogen was methicillin-sensitive Staphylococcus aureus (MSSA). Difficult-to-treat (DTT) infections and post-traumatic etiology were identified as predictors of treatment failure in patients undergoing two-stage TJR for hip and knee SA.

**Conclusions:**

Two-stage TJR in hip and knee SA demonstrated favorable clinical outcomes at mid-term follow-up. The procedure significantly improved functional scores and achieved a high success rate, while DTT infections and post-traumatic etiology were associated with a higher risk of treatment failure.

## Introduction

Septic arthritis (SA) is a rare but significant clinical challenge in orthopedics that may impact patients’ quality of life [1]. The SA annual incidence is estimated between 4 and 10 cases per 100,000 individuals and is increasing, mainly due to the aging population and the rising number of orthopedic procedures. Patients with diabetes, rheumatoid arthritis, immunocompromised status, underlying joint disease, or a history of previous intra-articular corticosteroid injections have an increased risk of developing SA [1, 2]. In most cases, SA presents as monoarticular, primarily affecting a single large joint such as the hip or knee. However, sometimes polyarticular septic arthritis may also occur, affecting multiple or smaller joints [3]. SAs are caused by pathogens’ joint invasion causing acute inflammation and progressive tissue destruction. Staphylococcus aureus is the most frequently identified; however, several bacteria, viruses, and fungi, carried through the bloodstream or spread locally from adjacent infected tissues, may be involved [4].

The SA diagnosis requires clinical evaluation, laboratory tests, imaging, and intraoperative findings. The typical joint clinical presentation is swelling, pain and warmth. Patients are usually unable to weight-bearing on the affected side and often describe pain during the active and passive joint excursion [3]. Serum laboratory evaluation for septic arthritis includes tests such as white blood cell (WBC) count, erythrocyte sedimentation rate (ESR), and C-reactive protein (CRP) level. Synovial fluid (SF) culture is the gold standard for diagnosis, but blood cultures are also recommended, especially when joint infection is likely to have spread through the bloodstream [3, 4]. Traditional criteria for diagnosis of SF were white blood cell count > 50,000 cells/mm^3^ or > 90% polymorphonuclear leukocytes, but they demonstrated low sensitivity and specificity. Recently, Varady et al. proposed the SF neutrophil-to-lymphocyte ratio as a simple and more accurate test to diagnose SA [5]. Imaging is performed to evaluate the joint and surrounding soft tissue damage. Radiographs may detect extensive cartilage erosion and intraarticular changes. Ultrasonography may reveal effusions. Magnetic resonance imaging (MRI) and computed tomography (CT) could have limited value for establishing the diagnosis in case of clinical suspicion. Still, they are sensitive in evaluating cartilage, bone, and soft tissue involvement and guiding the extent of debridement [3, 6]. Nuclear medicine, such as scintigraphy or positron emission tomography (PET), may be used as a third-level imaging technique. Still, they are not always available in every center and are characterized by high rates of false positives [7]. Finally, histological and microbiological analyses on intraoperative surgical samples are used to complete SA diagnosis [3, 4]. SA treatment requires a multidisciplinary approach involving orthopedics and infectious disease specialists. Arthroscopic irrigation, debridement, and antibiotics administration are minimally invasive options and may efficiently treat cases of early-stage SA [8–10]. In cases of severe joint degeneration, open approaches are indicated better to manage the debridement of infected tissues and consequent articular deformity. In 1943, Girdlestone described resection arthroplasty to treat hip SA; however, even if this technique demonstrated optimal infection control and pain relief, the residual loss of function is not considered acceptable by most patients [11, 12]. Total joint replacement (TJR) has emerged as a reliable treatment to control pain and infection, and to provide satisfactory articular function [13–15]. One-stage TJR has reported good functional outcomes but still has concerns regarding infection eradication [16–19]. Two-stage procedures with cement spacers, which locally elute antibiotics and maintain joint space, have shown excellent results regarding infection eradication and clinical-functional outcomes [15, 20–22]. The selection of treatment should also consider the nature of the infection, determining whether it is active or quiescent. Active infections, typified by an ongoing septic process, are generally indicated by positive clinical signs and laboratory tests. On the other hand, quiescent septic arthritis (SA) is characterized by a history of previously documented infection, but in the absence of any current positive clinical signs or laboratory test results [19, 23]. This study aims to examine the clinical outcomes of patients undergoing two-stage TJA in active hip and knee SA at a mid-term follow-up. Additionally, the study investigates the rates of complications, identifies the pathogens involved, and analyzes potential predictors of failure in the two-stage TJA process.

## Materials and methods

### Data collection and inclusion criteria

A retrospective analysis of the prospectively collected institutional arthroplasty registry was conducted from January 1st, 2012, to January 1st, 2019, searching for patients who received a two-stage TJR for hip or knee SA. Patients suffering from active hip or knee SA treated with two-stage replacement and at least two years of follow-ups were considered eligible for the study. Patients who had a two-stage procedure due to periprosthetic joint infection (PJI), patients with incomplete clinical-surgical data or were missing at the final follow-up were excluded.

### Diagnosis of SA

The diagnosis of SA was made based on the presence of one or a combination of the following parameters: clinical signs (edema, erythema, functional limitation, a draining sinus communicating with the joint), elevated erythrocyte sedimentation rate (ESR) > 30 mm/h, elevated serum C-reactive protein (CRP) > 5 mg/dL, positive cultures from arthrocentesis, radiographic findings of bone resorption and loss of articular space, intraoperative purulence, and intraoperative microbiology.

### Two-stage protocol

The same surgeon performed all the surgical procedures. Hips were operated through a posterolateral approach, and all the knees were operated using a medial parapatellar approach. During the first stage, femoral head and acetabulum or knee resection were performed, along with extensive debridement as previously described. Three to six samples of septic tissue were withdrawn for cultures. Preformed articulating antibiotic-loaded cement spacers containing gentamicin and vancomycin were used in all the cases included. The senior surgeon defined the bone defects using the Paprosky and the Anderson Orthopedic Research Institute (AORI) classifications [24–26] (Table 1). After the first stage, at least two weeks of broad-spectrum intravenous antibiotic therapy was administered. Then, the switch to targeted oral or intravenous antibiotic therapy for at least four weeks is based on the microbiological results.

**Table 1:** Classification of bone defects

		Hips (%)
**Paprosky** **acetabulum**	**I**	23 (37.7)
	**IIA**	16 (26.2)
	**IIB**	11 (18.0)
	**IIIA**	6 (9.8)
	**IIIB**	5 (8.2)
**Paprosky** **Femur**	**I**	48 (78.7)
	**II**	13 (21.3)
		***Knees (%)***
**AORI** **Femur**	**I**	21 (39.6)
	**IIA**	15 (28.3)
	**IIB**	9 (17.0)
	**III**	8 (15.1)
**AORI** **Tibia**	**I**	22 (41.5)
	**IIA**	16 (30.2)
	**IIB**	10 (18.9)
	**III**	5 (9.4)

AORI, Anderson Orthopaedic Research Institute

During the second stage, the spacer was explanted, and further debridement was performed. Three to six bioptic specimens were retrieved for microbiological analysis, as were specimens for frozen section histology. In case of suspected persistence of infection, a spacer exchange was performed.

After the second stage, antibiotic therapy was administered until the results of the intraoperative microbiology were received and continued after that when needed. Thromboembolism prophylaxis was started on day 0 with heparins and compressive stockings. Patients were encouraged to have partial weight-bearing starting from the second postoperative day.

### Data collection and outcome measures

Patients’ demographic characteristics and surgical variables (age, gender, body mass index [BMI], American Society of Anesthesiologists [ASA] physical status classification, type of pathogen involved, etiology of the infection, the level of bone defect, comorbidities, length of the interstage period) were collected from the institutional arthroplasty register, as were pre- and postoperative Knee Society Score (KSS), Harris Hip Score (HHS). When enterococci caused infections, methicillin/oxacillin, or vancomycin-resistant staphylococci; extended-spectrum beta-lactamase (ESBL)-producing bacteria; multidrug-resistant organisms (MDROs); and polymicrobial infections, they were classified as difficult-to-treat (DTT) infections. Treatment failure was defined as the recurrence of infection at any time during the two-stage protocol, spacer exchange, and cases of revision surgery after the reimplantation due to any cause.

### Statistical analysis

Categorical variables were expressed as the number of events or percentages. Continuous variables were expressed as the mean ± standard deviation (SD). Values of preoperative HHS and KSS were compared to those obtained at the final follow-up using the paired t-test. The distribution of these variables was evaluated using the Shapiro-Wilk test. Binomial logistic regression was performed to ascertain the effect of age, etiology, type of pathogen, ASA, and BMI on the risk of two-stage protocol failure. Kaplan–Meier curves were generated to analyze the failure probability distribution. Statistical analysis was conducted using IBM SPSS Statistics version 26.0 (IBM Corp., Armonk, NY, USA). For all the variables assessed, statistical significance was set at *p* < 0.05.

## Results

**Fig. 1:**
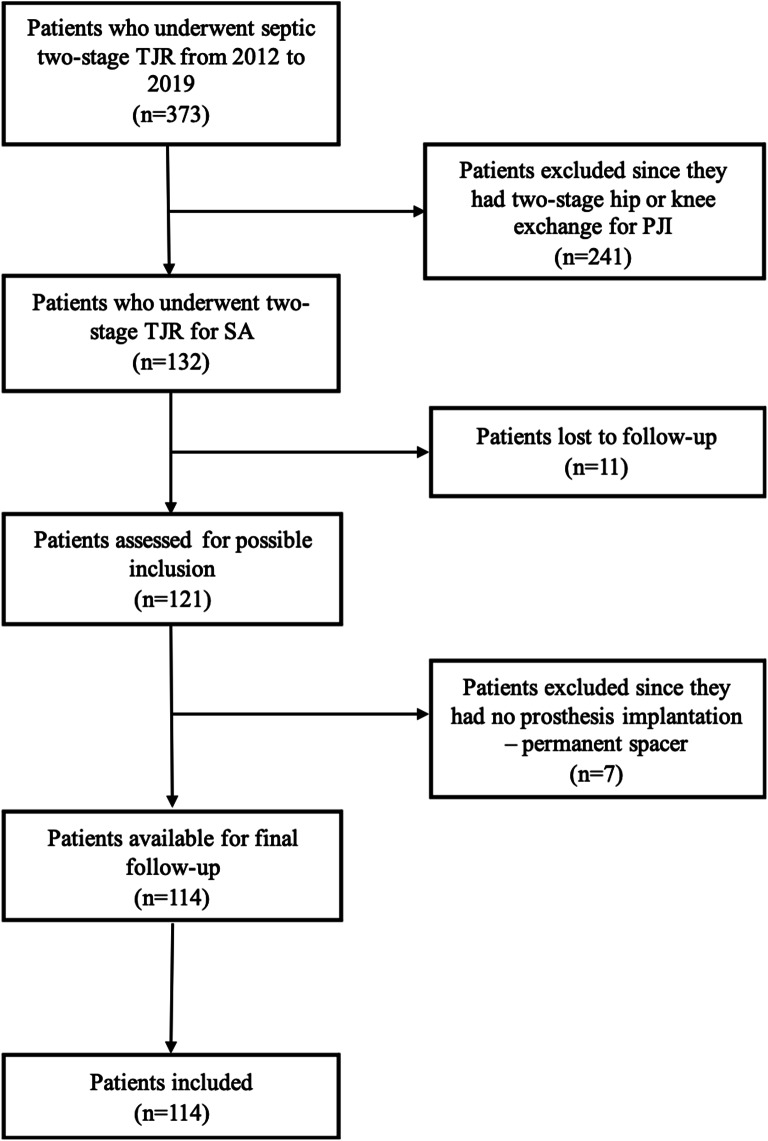
Flowchart of patients’ inclusion process. TJR: total joint replacement; PJI: periprosthetic joint infection; SA: Septic arthritis

One hundred and fourteen consecutive patients (60 men and 54 women) met the inclusion criteria and were enrolled in the study (Fig. 1). Of these, 61 (53.5%) had a hip SA, and 53 (46.5%) had a knee SA. The overall mean age at the second stage was 67.7 ± 9.1 years. The overall mean BMI was 26.7 ± 4.2 kg/m2. The overall mean time from stage one to reimplantation was 14.6 ± 2.8 weeks. The overall mean follow-up was 72.8 ± 22.9 months. The general characteristics of the patients included, and the main comorbidities registered are presented in Tables 2 and 3, respectively.

**Table 2:** Demographic characteristics of patients

Variables		Hips (%)	Knees (%)
**Sex M** **F**		34 (55.7) 27 (44.3)	26 (49.1) 27 (50.9)
**Mean age ± SD, y**		65.9 ± 9.5	67.5 ± 8.7
**Mean BMI (± SD), kg/m** ^**2**^		26.9 ± 4.3	26.4 ± 4.0
**ASA**	**I**	18 (29.5) 39 (64.0) 4 (3.5)	14 (26.4) 27 (50.9) 12 (22.6)
	**II**		
	**III**		
**Etiology**	**Post-surgery**	23 (37.7) 7 (11.4) 31 (50.8)	19 (35.8) 11 (20.8) 23 (42.6)
	**Post-infiltrative**		
	**Primary**		
**Mean interstage interval (± SD), w**		14.5 ± 2.9	14.7 ± 3.2
**Mean follow-up (± SD), m**		62.5 ± 28.6	72.8 ± 22.9

ASA, American Society of Anesthesiology; F, female; L, left; M, male; m, months; n, numbers; R, right; SD, standard deviation; y, years

**Table 3:** Main comorbidities

Condition	Hips (%)	Knees (%)
**DM**	22 (36.1)	19 (35.8)
**Drug abuse**	18 (29.5)	14 (26.4)
**HIV**	15 (24.6)	16 (30.2)
**HCV**	17 (27.9)	13 (24.5)
**Systemic TBC**	4 (6.6)	2 (3.8)
**CVD**	23 (37.7)	21 (39.6)
**CPD**	19 (31.1)	17 (32.1)

CPD, chronic pulmonary disease; CVD, cardiovascular disease; DM, diabetes mellitus; F, female; HCV, hepatitis C virus; HIV, human immunodeficiency virus; TBC, tuberculosis

### Clinical and functional outcomes

The mean HHS significantly improved from 39.7 ± 9.8 points preoperatively to 85.2 ± 10.2 points at the final follow-up (*p* < 0.001). The mean KSS improved from 40.6 ± 8.3 points preoperatively to 85.9 ± 8.4 points at the final follow-up (*p* < 0.001). The mean KSS function score improved from 24.7 ± 14.3 points to 84.2 ± 25.5 points (*p* < 0.001).

### Survival analysis

**Fig. 2:**
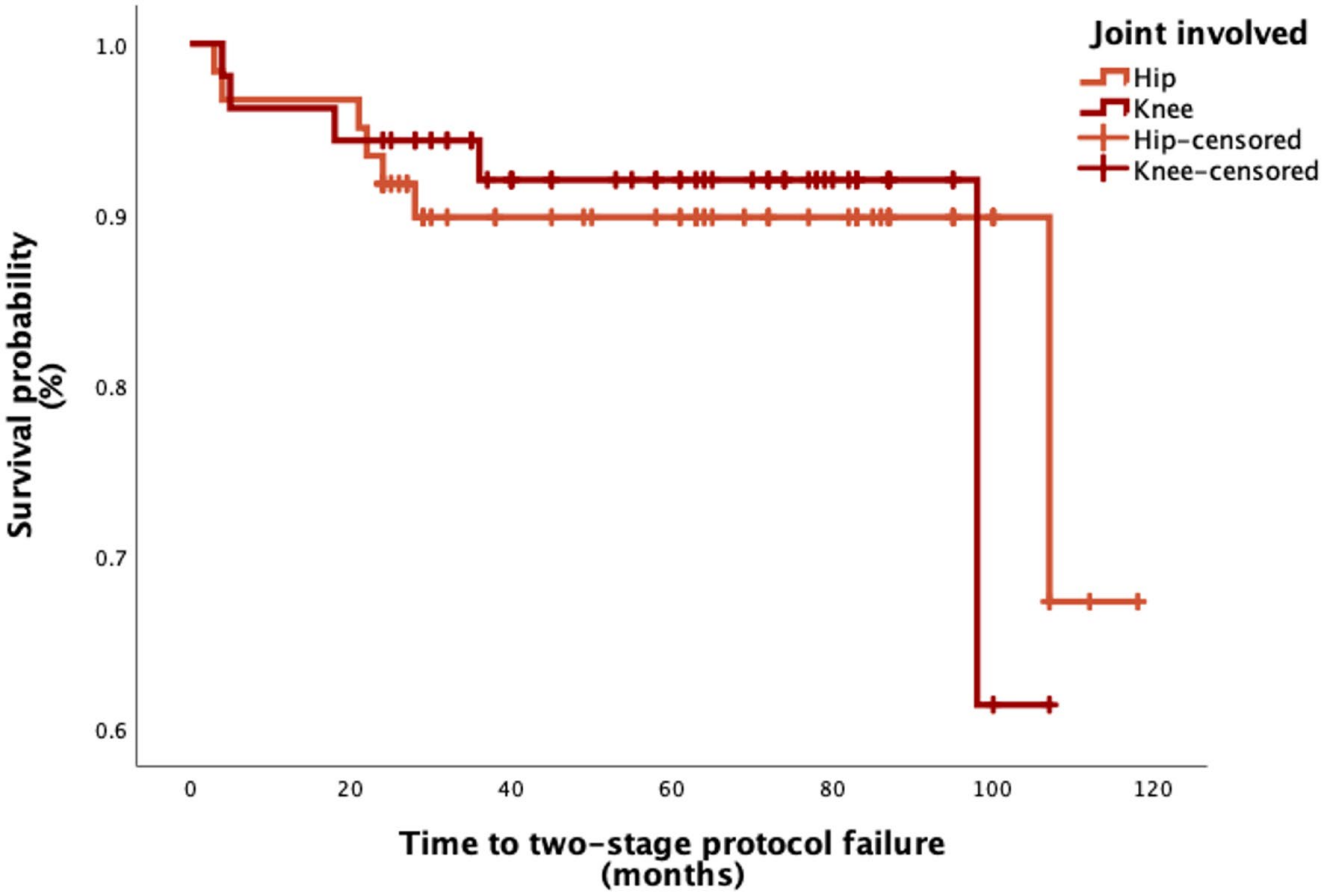
Kaplan-Meier curves investigating the success rate of the two-stage protocol for the treatment of septic arthritis

The overall success rate of the two-stage protocol was 89.5%. The success rate was 91.2% in patients who underwent hip surgery and 88.7% in patients who underwent knee operation. This difference was not statistically significant (*p* = 0.905). Figure 2 displays the Kaplan-Meier analysis performed. Treatment failures were due to the following causes: four patients (3.5%, two knees and two hips) had a recurrence of the infection during the interstage period and had a spacer exchange procedure. Three patients (2.6%, two hips and one knee) had a PJI after the second stage, which required revision, two patients (1.8%) who suffered SA of the knee had aseptic loosening of the implant, and two patients who had hip SA had recalcitrant infections that ultimately led to Girdlestone procedure.

### Complications

The overall rate of complications that did not require the revision of the prosthetic implants was 21%. Nine patients (7.9%, five hips and four knees) underwent spacer dislocation, which was managed through conversion to TJR. These patients demonstrated no further complications after spacer removal and prosthesis implantation at the final follow-up. Eleven patients (9.6%) developed heterotopic ossification of the hip, visible on radiographs. All the ossifications were non-progressive, asymptomatic, and no further treatment was indicated. Four patients (2.1%) who had hip two-stage arthroplasty developed hematoma after final implantation; in one case, surgical drainage of the hematoma was required.

### Pathogens

Methicillin-sensible Staphylococcus aureus (MSSA) was the most common pathogen in microbiology evaluation (19 cases, 16.7%). Methicillin-resistant Staphylococcus aureus (MRSA) was identified in four cases (20 cases, 17.5%), coagulase-negative staphylococci (CoN-St) in 15 patients (13.2%), and Mycobacterium tuberculosis. Polymicrobial and culture-negative infections were encountered in 16 (14.0%) and 23 (20.8%) cases, respectively. The complete list of pathogens identified is shown in Table 4.

**Table 4:** Pathogens involved in the infection

Pathogens	Hips (%)	Knees (%)
**MSSA**	11 (18.0)	8 (15.1)
**MRSA**	9 (14.8)	11 (20.8)
**CoN staphylococci**	6 (9.8)	9 (17.0)
***Streptococcus*** **sp.**	1 (1.6)	2 (3.8)
***Pseudomonas*** **sp.**	2 (3.2)	1 (1.9)
***Mycobacterium*** **sp.**	2 (3.2)	1 (1.9)
***Enterococcous*** **sp.**	3 (4.8)	2 (3.8)
***E. coli***	4 (6.4)	1 (1.9)
***Proteus*** **sp.**	2 (3.2)	-
**Polymicrobial**	9 (14.8)	7 (13.2)
**Culture negative**	12 (19.7)	11 (20.8)

MRSA, methicillin-resistant *Staphylococcus aureus*; MSSA, methicillin-sensible *Staphylococcus aureus*; CoN, coagulase-negative; sp., species

### Factors predicting the risk of two-stage failure

Of the six variables assessed, two significantly predicted the risk of treatment failure: the presence of DTT infections (OR = 7.4, 95% CI 1.5–37.9, *p* = 0.016) and the post-traumatic etiology of the infection (OR = 6.1, 95% CI 1.5–25.3, *p* = 0.013). This means that patients with DTT infections had a 7.4 times higher risk of treatment failure than patients without DTT infections, and patients who had a SA after internal fixation of previous fractures had a 6.1 times higher risk. The logistic regression model used was statistically significant χ2 (8) = 49.2, *p* < 0.001. The model explained 47.7% of the treatment failure variance and correctly classified 87.7% of the cases. Details are shown in Table 5.

**Table 5:** Logistic regression predicting the likelihood of two-stage revision failure based on age, ASA, BMI, type of infection, and extent of the bone defect

Variables	B	S.E.	p-value	Odds Ratio	95% C.I. for Odds Ratio	
					Lower	Upper
Age	− 0.017	0.040	. 676	0.676	0.984	0.910
ASA	− 0.320	0.664	0.630	0.726	0.198	2.668
BMI	0.320	0.664	. 630	. 686	0.686	0.492
Type of infection	2.005	0.833	. 016	7.426	1.452	37.988
Etiology	1.802	0.729	0.013	6.064	1.454	25.293
Level of bone defect	0.826	0.771	0.285	2.283	0.503	10.357

ASA, American Society of Anesthesiologists index; BMI, body mass index; S.E., standard error

*Note* Type of infection is for DTT infection compared to non-DTT infections. Etiology is for post-traumatic SA compared to post-infiltrative and primary SA

## Discussion

The main findings of the study were that, at mid-term follow-up, in patients with hip or knee SA treated with two-stage TJR, clinical and functional outcomes were significant statistical superior in the postoperative, with an overall success rate of 89.5% and an overall rate of complications that did not require revision of prosthetic implants of 21%. The pathogen mainly involved was MSSA, and it was reported that the presence of DTT pathogens and the post-traumatic infection etiology were related to a higher risk of two-stage TJR treatment failure.

SA of the native joint, either knee or hip, is relatively uncommon and requires prompt diagnosis and treatment [1, 2]. The diagnosis might be challenging. The most common cause of adult SA is occult bacteremia which seeds a joint synovium. Staphylococcus aureus is the most detected causative agent isolated in 36–57% of patients [2, 3]. Several risk factors have been described, including age older than 60 years, diabetes mellitus, recent bacteremia, malignancy, hepatic cirrhosis, nephropathy, drug or alcohol abuse, a history of corticosteroid injection, a recent injury or surgical procedure, and rheumatoid arthritis [1, 4]. The anamnesis associated with the clinical presentation should lead to the suspicion of SA. Successively, laboratory and SF analyses are the most important diagnostic tools to detect SA [6]. However, a negative SF culture does not exclude the possibility of SA [13, 14]. Imaging is relatively helpful in the diagnosis. At the same time, it provides information on the joint and the surrounding tissue, which is of special interest in chronic SA. An early diagnosis is an important prognostic factor associated with better outcomes. Although guidelines exist, there is a lack of international consensus on the proper management of SA [8, 27]. Arthroscopic debridement, arthrotomy, and one or two-stage total joint arthroplasty are recommended in SA [6, 9, 15]. Given its reduced invasiveness, arthroscopy is preferred for acute SA, reporting similar outcomes compared to joint arthrotomy in patients with non-MRSA pathogens [2, 3]. Resection arthroplasty was first described by Girdlestone [28]. However, limb length discrepancy, limited mobility and bearing occur [12, 29]. One or two-stage arthroplasty provides good long-time outcomes [10, 14, 20]. Although one-stage arthroplasty after SA is reliable, concerns about periprosthetic joint infection exist, especially if the infection is not completely eradicated [16, 30, 31]. Bettencourt et al. [17] reported a six-fold increased risk of periprosthetic joint infection in patients undergoing total knee arthroplasty with a history of native knee SA compared to controls undergoing the same procedure for osteoarthritis, with a cumulative incidence of 9% at ten years [17]. Portier et al. [18] reported in their retrospective study of 49 arthroplasties for SA an incidence of 10% of the development of periprosthetic joint infection [18]. One-stage arthroplasty should be performed only in patients with a previous SA quiescent at the time of surgery [19]. On the other hand, the two-stage arthroplasty approach is characterized by the temporary implantation of antibiotic-loaded or not cement beads and/or a static or articulating spacer, followed by the ultimate arthroplasty [32, 33]. The joint spacer allows a better function, as it maintains the length and rotation of the joint, and when antibiotics-loaded, it has a microbicidal effect [34]. Antibiotic-loaded-spacers effectively eradicate joint infection and promote good long-term outcomes [10, 14, 20]. Unfortunately, spacer-related complications are common, with a prevalence of 26-58.5% of patients after staged revision arthroplasty [35]. The spacer may break, dislocate, or lead to bone loss, which could impact the prosthesis implantation [21]. In a previous study on 139 patients, infection-free survival was evidenced in 95.6% of patients at approximately four years of follow-up [21]. The reported reinfection rate was 4.4.% at two years. Previous investigations reported a higher incidence of reinfection rate [21, 36]. The two-stage procedures have reported a high eradication rate following the second-step surgery, ranging from 85 to 100% [13, 14, 16]. Moreover, Russo et al. [22] conducted a single-center retrospective study on two-stage total hip or knee arthroplasty in SA with a minimum five-year follow-up, and 47 patients were enrolled. They reported a significant improvement in functional scores and an overall eradication rate of 93.6% [22]. In another review [15] on two-stage arthroplasty for SA of the hip and knee on 21 studies (435 procedures), the authors found a significant improvement in the functional outcome at a mean of 53.7 months of follow-up [15]. The complication rate was 20.2%; of them, 4.1% were septic recurrences during the interstage period successfully treated with spacer exchange, and 3.6% were spacer-related complications [15]. Overall, the mean infection eradication was 93.3% [15], demonstrating that the two-stage approach is a reliable and high-efficacy treatment for managing SA patients. The logistic regression model used, which considered six variables, reported a statistically significant higher risk of TJR failure in SA associated with DTT infections and post-traumatic etiology. As described by Wei et al. in their study [37], more virulent and atypical pathogens are often associated with complex and sometimes inadequate antibiotic treatment that is unable to eradicate the infection at the involved joint. In post-traumatic or after internal fixation surgery SA, an increased risk of failure may be associated due to poor local tissue condition after repeated surgeries [37]. Moreover, internal fixation devices can be hard to remove, and remnants of septic material can drive recurrence of infection. Another issue that must be considered when dealing with post-traumatic SA is the possible presence of malunions or axial deformities, which can ultimately have an impact on prosthesis fixation, balance, and long-term survivorship. To the best of our knowledge no paper in the literature has already assessed which factors could lead to failure of two-stage TJR for SA. The findings of this study should be kept in considerations by surgeons treating SA, to have realistic expectations on treatment outcomes and to better counsel patients who have such risk factors.

The present study has several strengths and limitations to consider. The main strength is including a large sample of patients with septic hip or knee infections treated with two-stage TJR operated by a single surgeon in a standardized fashion, then providing valuable insights at mid-term follow-up. However, there are also several limitations. Firstly, the study’s retrospective design introduces the possibility of selection bias and incomplete data collection. Secondly, the study was conducted in a single center, potentially limiting the generalizability of the findings to other institutions or populations. Third, the study lacks a comparison with alternative treatment options for SA, thereby limiting the assessment of the relative efficacy of the two-stage TJR method compared to various treatment strategies. Fourth, information on previous attempts to treat the infection was not always available for all patients. For this reason, the type of primary treatment (open or arthroscopic) was not included as a variable in the statistical analysis, even if it must be recognized that it could impact the success rate of the two-stage protocol. Fifth, hips and knees were analyzed together, and a logistic regression model was built considering a mixed hip and knee cohort. Unfortunately, the relatively small sample size of the study did not allow us to perform the analysis separately based on the joint involved. Lastly, although a mid-term follow-up was performed, a detailed analysis of long-term outcomes was not provided, limiting the understanding of the long-term effectiveness of the two-stage TJR procedure. Longer-term and prospective studies are needed to confirm the results of this study and evaluate the long-term efficacy of the two-stage TJR procedure in the treatment of SA of the hip and knee.

## Conclusion

Management of hip and knee SA remains a complex clinical challenge. DTT pathogens and a post-traumatic etiology of infection were significantly associated with an increased likelihood of treatment failure in patients undergoing two-stage TJR for hip and knee SA. These factors emerged as predictors of unfavorable outcomes, underscoring the importance of careful consideration and proactive management when encountering such cases. A prompt diagnosis, appropriate treatment, and a multidisciplinary approach are crucial to achieve favorable outcomes and minimize complications. TJR has emerged as a reliable solution for patients with SA and extensive joint degeneration, offering symptom control and functional restoration.

## Data Availability

The dataset analysed in this study is available from the corresponding author on reasonable request.
